# Supplementary material for: The effects of locomotion on sensory-evoked haemodynamic responses in the cortex of awake mice
Source: Sci Rep. 2022 Apr 14;12:6236. doi: 10.1038/s41598-022-10195-y (PMC9010417; doi:10.1038/s41598-022-10195-y)
Supplement: Supplementary file 1 — Supplementary Information. [file 41598_2022_10195_MOESM1_ESM.pdf]

Supplementary figures and tables

The effects of locomotion on sensory-evoked haemodynamic responses in the cortex of awake mice

Beth Eyre[1,2,3^*], Kira Shaw[1,5^], Paul Sharp[1,6], Luke Boorman[1], Llywelyn Lee[1,2,3], Osman Shabir[1,2,3,4], Jason Berwick[1,2,3+], Clare Howarth[1,2,3+]

[1]Sheffield Neurovascular Lab, Department of Psychology, University of Sheffield, Alfred Denny Building, Western Bank, Sheffield, S10 2TN (United Kingdom)

[2]Neuroscience Institute, University of Sheffield, Sheffield, S10 2TN (United Kingdom)

[3]Healthy lifespan Institute, University of Sheffield

[4]Department of Infection, Immunity & Cardiovascular Disease (IICD), University of Sheffield Medical School, Royal Hallamshire Hospital, Beech Hill Road, Sheffield, S10 2RX (United Kingdom)

[5]School of Psychology and Sussex Neuroscience, University of Sussex, Falmer, Brighton, United Kingdom

[6]Medicines Discovery Catapult, Mereside, Alderley Park, Alderley Edge, SK10 4TG

[^]These authors contributed equally. [+]These authors contributed equally. * Corresponding author.

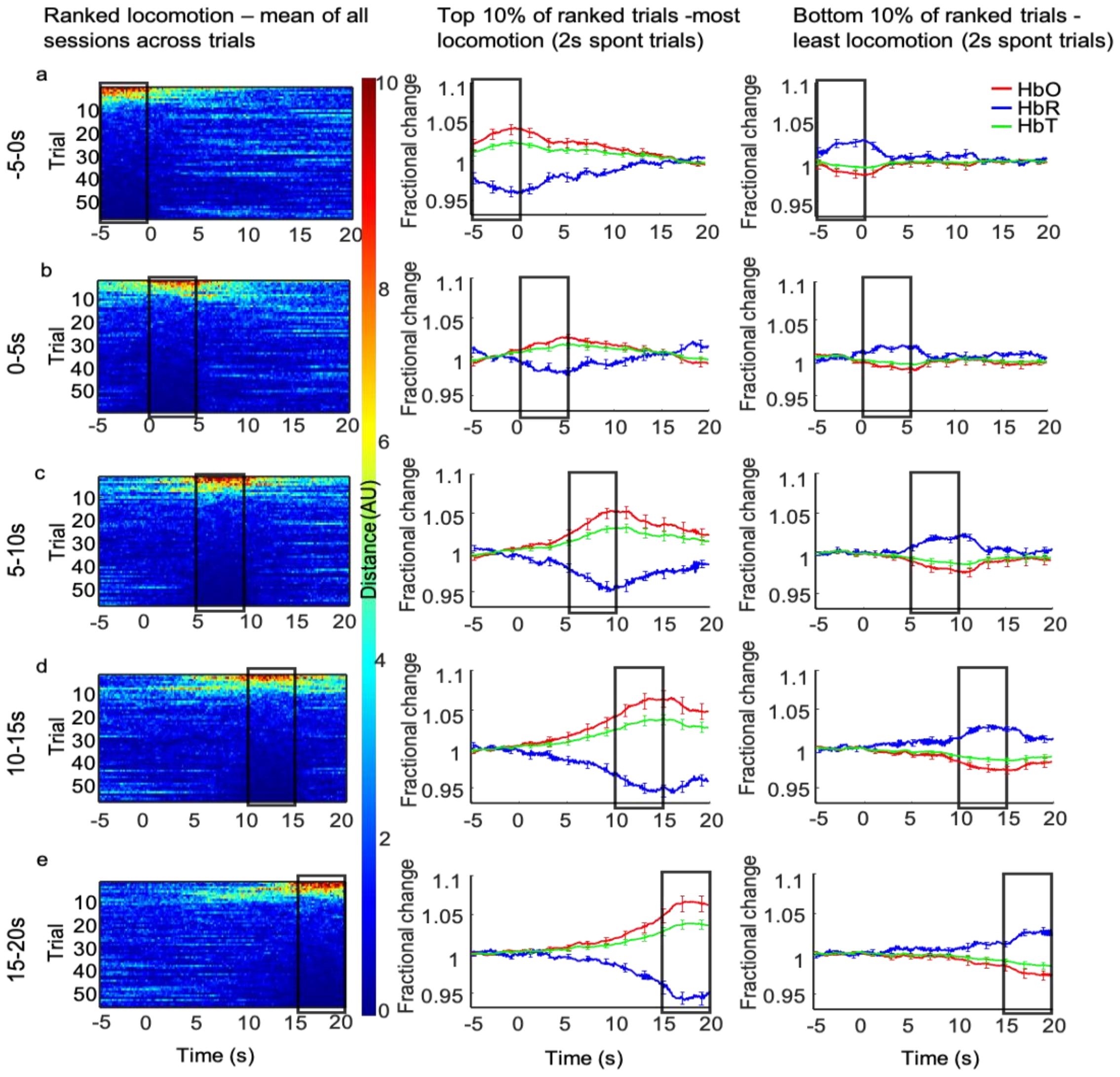

Supplementary figure 1: Mean locomotion induced haemodynamic responses for the whisker ROI (Fig. 5 column 1) during trials where the most and least locomotion occurred with locomotion ranked at different time windows throughout a 25s time period. Column one: heat maps show locomotion traces for the 59 whisker-stimulation trials, with spontaneous locomotion ranked at different 5s time windows during a 25s time period (each ranked trial was averaged across 21 sessions/4 animals). The different 5s windows during the 25s time period where locomotion was ranked are: before (**a:** -5-0s), during (**b:** 0-5s) and after whisker stimulation (**c:** 5-10s, **d:** 10-15s, **e:** 15-20s). Trials were ranked according to locomotion in these 5s periods and presented in descending order. Colour bar indicates amount of locomotion, red pixels indicate more locomotion and dark blue indicate less locomotion. **Column two:** mean fractional changes from baseline in HbT, HbO and HbR taken from the top 10% of ranked locomotion trials across the different 5s time windows (21 sessions/4 animals; n=6 top per session (mean of top trials taken for each session, mean of all sessions used in the visualisation/analysis). **Column three:** mean fractional changes in HbT, HbO and HbR for the bottom 10% of locomotion trials ranked across the 5s time windows throughout the trial (21 sessions/4 animals; n=6 bottom per session (mean of bottom trials taken for each session, mean of all sessions used in the visualisation/analysis). Black boxes indicate the 5s time window locomotion was ranked. Data show mean across the total 126 trials +/- SEM.

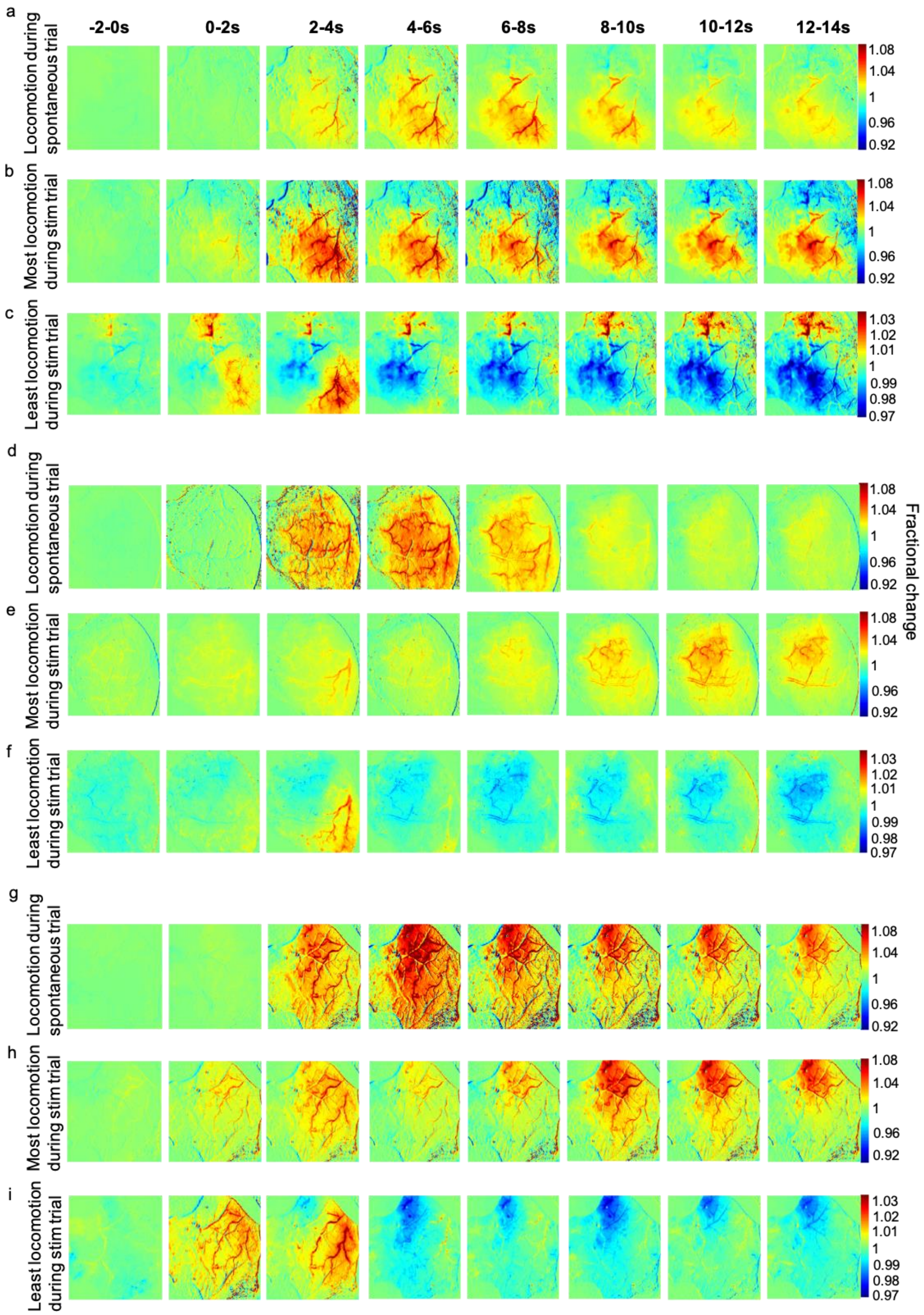

a
b
c
d
e
f
g
h
i
-2-0s
0-2s
2-4s
4-6s
6-8s
8-10s
10-12s
12-14s
Locomotion during spontaneous trial
Most locomotion during stim trial
Least locomotion during stim trial
1.08
1.04
1
0.96
0.92
1.03
1.02
1.01
0.99
0.98
0.97
Fractional change

Supplementary figure 2: HbT spatial montages for each animal during spontaneous locomotion and most and least locomotion during a 2s whisker stimulation. HbT spatial maps reveal fractional change in HbT across the vasculature over time, starting 2s prior to whisker stimulation (or locomotion) to 14s post stimulation. Each spatial image is an average of 2s of data. Whisker stim/spontaneous locomotion occur at 0s. Red pixels indicate an increase in activation and blue pixels indicate a decrease in activation. **(a-c)** show spatial HbT maps for animal 1 for spontaneous locomotion **(a),** most locomotion during a 2s whisker stim **(b)** and least locomotion during a 2s whisker stim **(c)**. **(d-f)** show the HbT spatial responses for animal 3 for spontaneous locomotion **(d),** most locomotion during a 2s whisker stim **(e)** and least locomotion during a 2s whisker stim **(f). (g-i)** show the HbT spatial responses for animal 4 for spontaneous locomotion **(g),** most locomotion during a 2s whisker stim **(h)** and least locomotion during a 2s whisker stim **(i)**.

Supplementary tables for statistics outputs

All data values were included in the statistical analysis even if outliers were found as there was no scientific reason to remove them from the data set. Data used in the analysis can be found in the DRYAD repository, doi:10.5061/dryad.v41ns1rxs

<u>Distance travelled</u>

Supplementary table S1: Shapiro Wilk test of normality for distanced travelled during spontaneous and whisker stimulation trials

Distance travelled	Statistic	df	p
Spontaneous vs Whisker stim	.691	21	.000

Normality was assessed using a difference variable comparing distanced travelled during spontaneous trials with distance travelled during whisker stimulation trials

Supplementary table S2: Sign test result for distance travelled during spontaneous and whisker stimulation trials

Type of trial	N	Mean	Median	SEM	z	p
Spontaneous	21	13610	12360	1532	-	-
2s whisker stim	21	17802	14894	2732	-	-
Difference	21	-4192	-1517	2065	1.309	.189

Greatest vs Least locomotion

Supplementary table S3: Shapiro Wilk test of normality for greatest vs least locomotion (where locomotion ranked across the entire 25s trial)

	Greatest Locomotion vs Least Locomotion		
**Haemodynamic Measure**	**Statistic**	**df**	**p**
HbT	.568	21	.000
HbO	.695	21	.000
HbR	.923	21	.100

Supplementary table S4: Wilcoxon signed ranks result for HbR greatest vs least locomotion (where locomotion is ranked across the entire 25s trial)

	N	Mean	Median	SEM	z	p
HbR peak greatest locomotion	21	0.947	0.948	.006	-	-
HbR peak least locomotion	21	0.947	0.945	.005	-	-
Difference most vs least HbR	21	0.000	-.0004	.005	-.330	.741

Supplementary table S5: Sign test result for HbT and HbO greatest vs least locomotion (where locomotion is ranked across the entire 25s trial)

	N	Mean	Median	SEM	z	p
HbT peak greatest locomotion	21	1.043	1.038	.007	-	-
HbT peak least locomotion	21	1.030	1.028	.003	-	-
Difference most vs least HbT	21	.013	.003	.007	-.873	.383
HbO peak greatest locomotion	21	1.065	1.057	.009	-	-
HbO peak least locomotion	21	1.053	1.049	.005	-	-
Difference most vs least HbO	21	.012	-.0004	.008	.000	1.000

Return to baseline

Supplementary table S6: Shapiro Wilk test of normality for baseline statistics (mean values taken from last 5s of the 2s whisker stimulation trial (between 15-20s)

Haemodynamic Measure	Statistic	df	p
HbT	.877	21	.013
HbO	.932	21	.152
HbR	.885	21	.018

Supplementary table S7: Sign test result for HbT and HbR return to baseline (HbT/HbR mean values between 15-20s for trials with the most locomotion compared with the mean values between 15-20s for trials with the least locomotion)

	N	Mean	Median	SEM	z	p
HbT most locomotion	21	1.018	1.019	.005	-	-
HbT least locomotion	21	0.995	0.997	.002	-	-
Difference HbT most vs least	21	.024	.024	.005	-3.055	.001
HbR most locomotion	21	0.982	0.980	.004	-	-
HbR least locomotion	21	1.000	0.999	.003	-	-
Difference HbR most vs least	21	-.022	-.021	.005	3.491	.000

Supplementary table S8: Wilcoxon Signed Ranks test for HbO return to baseline (HbO mean value between 15-20s for trials with the most locomotion compared with the mean value between 15-20s for trials with the least locomotion)

	N	Mean	Median	SEM	z	p
HbO most locomotion	21	1.028	1.030	.006	-	-
HbO least locomotion	21	0.992	0.994	.003	-	-
Difference HbO most vs least	21	.036	.032	.007	-3.702	.000

Two-way Repeated measures ANOVAs for 2s whisker simulation trials

Supplementary table S9: Shapiro Wilk test of normality for each time window, amount of locomotion and haemodynamic measures

	**Most Locomotion**						**Least Locomotion**					
	**HbT**		**HbO**		**HbR**		**HbT**		**HbO**		**HbR**	
**Time window**	df	p	df	p	df	p	df	p	df	p	df	p
-5-0	21	.000*	21	.001*	21	.518	21	.380	21	.174	21	.867
0-5	21	.000*	21	.001*	21	.654	21	.872	21	.680	21	.186
5-10	21	.000*	21	.000*	21	.429	21	.172	21	.234	21	.649
10-15	21	.000*	21	.001*	21	.316	21	.123	21	.496	21	.991
15-20	21	.000*	21	.005*	21	.843	21	.926	21	.739	21	.046*

*Performed on studentised residuals. Time window is the time window in which locomotion was calculated for ranking purposes. *Indicates normality is violated*

Supplementary table S10: Sphericity Results for the two-way repeated measures ANOVAs (Mauchly's test)

	HbT				HbO				HbR			
**Factor**	X^2	df	p	ε	X^2	df	p	ε	X^2	df	p	ε
Time	20.297	9	.017	.731	15.859	9	.071	.753	5.798	9	.761	.883
Time * Locomotion	24.286	9	.004	.645	17.771	9	.039	.723	20.637	9	.015	.632

ε indicates Greenhouse Geiser value. X^2 Chi–squared value. Values <.05 sphericity violated

Supplementary table S11: Two-Way Repeated ANOVA outputs for HbT, HbO and HbR investigating the interaction between the amount of locomotion (factors: most & least) and the time at which locomotion occurred (factors: -5-0s, 0-5s, 5-10s, 10-15s, 15-20s)

	HbT			HbO			HbR		
**Factor**	df	F	p	df	F	p	df	F	p
Time	2.925, 58.492	7.849	.000	4, 80	8.435	.000	4, 80	9.134	.000
Locomotion	1, 20	6.032	.023	1, 20	4.868	.039	1, 20	3.723	.068
Time * Locomotion	2.578, 51.566	13.351	.000	2.894, 57.876	13.317	.000	2.526, 50.521	8.712	.000

Supplementary table S12: Sphericity Results (Mauchly's test) for simple main effects (One-way ANOVAs) investigating the effects of timing of locomotion on sensory-evoked hemodynamic responses for the most and least locomotion

	HbT				HbO				HbR			
	X^2	df	p	ε	X^2	df	p	ε	X^2	df	p	ε
**Most**												
Time	29.923	9	.000	.630	30.653	9	.000	.665	19.251	9	.024	.672
**Least**												
Time	17.153	9	.047	.735	11.929	9	.219	.772	13.192	9	.156	.729

ε indicates Greenhouse Geiser value. Most refers to most locomotion, least refers to least locomotion

Supplementary table S13: Simple main effects to investigate the interaction between the amount and timing of locomotion and the effect on the haemodynamic response

Factor: Time	HbT			HbO			HbR		
	df	F	p	df	F	p	df	F	p
**Most Locomotion**	2.520, 50.409	12.998	.000	2.660, 53.192	12.788	.000	2.689, 53.781	11.504	.000
**Least Locomotion**	2.940, 58.791	8.933	.000	4, 80	9.939	.000	4, 80	6.829	.000

*One-way ANOVAs were used to assess simple main effects

Supplementary table S14: Notable comparisons between 5s time windows for HbT, HbO and HbR peaks during whisker stimulation during most or least locomotion conditions

Haemodynamic Measure	Time window for ranking locomotion and time window compared to	P value
**Most Locomotion**		
HbT	-5-0 > 15-20	.013
	0-5 > 5-10	.006
	0-5 > 10-15	.003
	0-5 > 15-20	.000
HbO	-5-0 > 10-15	.048
	-5-0 > 15-20	.008
	0-5 > 5-10	.008
	0-5 > 10-15	.003
	0-5 > 15-20	.000
HbR	-5-0 < 5-10	.017
	-5-0 < 10-15	.012
	-5-0 < 15-20	.005
	0-5 < 5-10	.013
	0-5 < 10-15	.004
	0-5 < 15-20	.001
**Least Locomotion**		
HbT	-5-0 > 0-5	.000
	0-5 < 5-10	.013
	0-5 < 10-15	.009
	0-5 < 15-20	.005
HbO	-5-0 > 0-5	.000
	0-5 < 5-10	.006
	0-5 < 10-15	.001
	0-5 < 15-20	.002
HbR	-5-0 < 0-5	.008
	0-5 > 5-10	.018
	0-5 > 10-15	.007
	0-5 > 15-20	.015

> Indicates greater than and < indicates less than

Supplementary table S15: Pairwise comparisons with a Bonferroni correction comparing mean peaks for trials in which most and least locomotion occurred during the different time windows

		**Most Locomotion**		**Least Locomotion**				
**Time window**	Measure	*M*	*SEM*	*M*	*SEM*	*df*	*F*	*p*
-5-0	HbT	1.049	± .008	1.035	± .003	1, 20	3.466	.077
	HbO	1.078	± .011	1.061	± .006	1, 20	2.401	.137
	HbR	0.930	± .008	0.944	± .006	1, 20	2.149	.158
0-5	HbT	1.054	± .007	1.020	± .002	1, 20	19.676	.000*
	HbO	1.083	± .009	1.033	± .004	1, 20	24.825	.000*
	HbR	0.934	± .006	0.969	± .004	1, 20	38.753	.000*
5-10	HbT	1.041	± .007	1.031	± .003	1, 20	3.144	.091
	HbO	1.063	± .009	1.052	± .005	1, 20	1.702	.207
	HbR	0.948	± .005	0.952	± .005	1, 20	.486	.494
10-15	HbT	1.037	± .005	1.034	± .004	1, 20	.756	.395
	HbO	1.058	± .007	1.057	± .006	1, 20	.057	.814
	HbR	0.951	± .005	0.948	± .006	1, 20	.316	.580
15-20	HbT	1.032	± .004	1.031	± .003	1, 20	.058	.812
	HbO	1.052	± .006	1.052	± .005	1, 20	.000	.995
	HbR	0.954	± .005	0.951	± .005	1, 20	.219	.645

**Indicates a significant difference between the mean peak of the haemodynamic measure when comparing most and least locomotion, with Bonferroni adjustment applied. M indicates mean and SEM indicates standard error of the mean*

Two-way Repeated measures ANOVAs for linear subtraction of spontaneous trials from 2s whisker stimulation trials

Supplementary table S16: Shapiro Wilk test of normality for each time window, amount of locomotion and haemodynamic measures

	Most Locomotion						Least Locomotion					
	**HbT**		**HbO**		**HbR**		**HbT**		**HbO**		**HbR**	
**Time window**	df	p	df	p	df	p	df	p	df	p	df	p
-5-0	21	.000*	21	.005*	21	.150	21	.742	21	.999	21	.923
0-5	21	.000*	21	.014*	21	.618	21	.665	21	.625	21	.036*
5-10	21	.007*	21	.374	21	.663	21	.003*	21	.030*	21	.132
10-15	21	.013*	21	.721	21	.832	21	.002*	21	.336	21	.402
15-20	21	.024*	21	.945	21	.410	21	.006*	21	.021*	21	.054

*Performed on studentised residuals. Time window is the time window in which locomotion was calculated for ranking purposes. *Indicates normality is violated*

Supplementary table S17: Sphericity Results for the two-way repeated measures ANOVAs (Mauchly's test)

	**HbT**				**HbO**				**HbR**			
**Factor**	X^2	df	p	ε	X^2	df	p	ε	X^2	df	p	ε
Time	15.667	9	.075	.707	14.081	9	.121	.733	8.073	9	.529	.798
Time * Locomotion	24.965	9	.003	.650	17.410	9	.043	.720	14.000	9	.124	.793

ε indicates Greenhouse Geiser value. X^2 Chi–squared value. Values <.05 sphericity violated

Supplementary table S18: Two-Way Repeated ANOVA outputs for HbT, HbO and HbR investigating the interaction between the amount of locomotion (factors: most & least) and the time at which locomotion occurred (factors: -5-0s, 0-5s, 5-10s, 10-15s, 15-20s)

	**HbT**			**HbO**			**HbR**		
**Factor**	df	F	p	df	F	p	df	F	p
Time	4,80	1.241	.300	4,80	0.622	.648	4,80	2.563	.045
Locomotion	1,20	0.426	.522	1,20	0.417	.526	1,20	2.030	.170
Time * Locomotion	2.599,51.984	5.290	.004*	2.878, 57.562	5.248	.003	4,80	3.123	.019

Supplementary table S19: Sphericity Results (Mauchly's test) for simple main effects (One-way ANOVAs) investigating the effects of timing of locomotion on sensory-evoked hemodynamic responses for the most and least locomotion

	HbT				HbO				HbR			
	X^2	df	p	ε	X^2	df	p	ε	X^2	df	p	ε
**Most**												
Time	23.435	9	.005	.679	19.677	9	.020	.708	15.675	9	.075	.687
**Least**												
Time	24.856	9	.003	.593	16.553	9	.057	.682	6.793	9	.660	.857

ε indicates Greenhouse Geiser value. Most refers to most locomotion, least refers to least locomotion

Supplementary table S20: Simple main effects to investigate the interaction between the amount and timing of locomotion and the effect on the haemodynamic response

**Factor: Time**	**HbT**			**HbO**			**HbR**		
	df	F	p	df	F	p	df	F	p
**Most Locomotion**	2.717, 54.340	3.915	.016	2.832, 56.646	2.891	.046	4,80	0.601	.663
**Least Locomotion**	2.373, 47.461	3.754	.024	4,80	4.179	.004	4,80	4.925	.001

*One-way ANOVAs were used to assess simple main effects

Supplementary table S21: Notable comparisons between 5s time windows for HbT, HbO and HbR peaks during whisker stimulation during most or least locomotion conditions

**Haemodynamic Measure**	**Time window for ranking locomotion and time window compared to**	**P value**
**Most Locomotion**		
HbT	0-5 > 15-20	.049
**Least Locomotion**		
HbT	0-5 < 10-15	.015
HbO	0-5 < 10-15	.006
HbR	0-5 > 10-15	.034

> Indicates greater than and < indicates less than

Supplementary table S22: Pairwise comparisons with a Bonferroni correction comparing mean peaks for trials in which most and least locomotion occurred during the different time windows

		Most Locomotion		Least Locomotion				
**Time window**	Measure	*M*	*SEM*	*M*	*SEM*	*df*	*F*	*p*
-5-0	HbT	1.034	± .007	1.040	± .004	1, 20	0.777	.388
	HbO	1.051	± .008	1.069	± .007	1, 20	3.198	.089
	HbR	0.948	± .007	0.929	± .007	1, 20	3.558	.074
0-5	HbT	1.046	± .007	1.026	± .003	1, 20	10.049	.005*
	HbO	1.070	± .009	1.046	± .004	1, 20	6.790	.017*
	HbR	0.943	± .006	0.953	± .005	1, 20	1.757	.200
5-10	HbT	1.037	± .006	1.035	± .005	1, 20	0.225	.640
	HbO	1.056	± .008	1.058	± .007	1, 20	0.090	.767
	HbR	0.951	± .006	0.948	± .005	1, 20	0.171	.683
10-15	HbT	1.034	± .004	1.038	± .004	1, 20	1.217	.283
	HbO	1.053	± .006	1.065	± .006	1, 20	3.969	.060
	HbR	0.950	± .006	0.934	± .006	1, 20	6.060	.023*
15-20	HbT	1.031	± .003	1.033	± .003	1, 20	0.735	.402
	HbO	1.051	± .005	1.058	± .005	1, 20	1.324	.263
	HbR	0.947	± .006	0.939	± .005	1, 20	1.334	.262

**Indicates a significant difference between the mean peak of the haemodynamic measure when comparing most and least locomotion, with Bonferroni adjustment applied. M indicates mean and SEM indicates standard error of the mean*
